# Engagement of African Americans with Rapid HIV Testing and HIV Care

**Published:** 2017-09-22

**Authors:** Safiya George Dalmida, Graham J. McDougall, George C. T. Mugoya, Pamela Payne Foster, Makenzie Plyman, Joe Burrage

**Affiliations:** 1 Capstone College of Nursing, University of Alabama, 650 University Blvd, Tuscaloosa, AL 35487-0358, USA; 2 Educational Studies in Psychology, Research Methodology and Counseling, The University of Alabama, College of Education, Box 870231, Tuscaloosa, AL 35487-0231, USA; 3 College of Community Health Sciences, University of Alabama, 850 5^th^ Avenue East Tuscaloosa, AL 35401, USA

**Keywords:** HIV, HIV testing, African American, HIV care, Disparities

## Abstract

**Introduction/Background:**

African Americans and Blacks experience the greatest human immunodeficiency virus (HIV) burden of any racial group in the US and globally. A number of challenges contribute to the higher rates of HIV infection among African Americans, including a lack of awareness of HIV status. African Americans account for nearly 50% of the newly estimated HIV/acquired immunodeficiency syndrome (AIDS) diagnoses, with the majority being tested only after developing symptoms of AIDS. Moreover, African Americans are more likely to postpone medical care after finding out that they are HIV positive.

**Purpose:**

The aim of this study was to describe African Americans’ likelihood of using salivary rapid testing (SRT) methods and entry into healthcare if HIV positive.

**Methods/Design:**

Focus groups were conducted among 38 African Americans. The purpose of this study was to (1) describe personal factors, social resources, socio-demographic factors, cognitive appraisal, and health and coping behaviors which predict or influence the likelihood of African Americans’ participation in SRT and, if positive, subsequent entry into healthcare and (2) to evaluate HIV Testing Survey items and modify them to be culturally and linguistically appropriate. A modified Comprehensive Health Seeking and Coping Paradigm guided the study (CHSCP).

**Results:**

Of the 38 African American adults who participated in the study, 16 were female between the ages of 18–49 and the mean age was 23 years and there were 22 males, aged between 18–49 and the mean age was 29.5 years. Eight themes emerged from the data: *familiarity*, *stigma*, *fear*, *access*, *immediacy*, *ease*, *degree of responsibility*, and *trust*. Gender specific themes were *health maintenance* (women) and *illness management* (men). Sub-themes within gender-specific themes were *stoicism* (women) and *anger* (men).

**Implications:**

Identifying the factors that influence the likelihood of HIV testing uptake can provide information on which to base development of interventions to facilitate HIV testing and earlier linkage to healthcare.

## INTRODUCTION

Over three decades after the discovery of human immunodeficiency virus (HIV), the continued spread of HIV infection in the United States remains a major concern. There are over 1.2 million individuals in the United States (U.S.) living with acquired immunodeficiency syndrome (AIDS) and approximately 50,000 people are newly diagnosed with HIV annually.^1^ African Americans experience the greatest HIV burden of any racial group in the U.S.^1,2^ For instance, in 2014 while African Americans represented about 12% of the U.S. population, they accounted for 44% of the new infections in the same year.^1^

HIV testing is a key first step to entry into HIV care, yet individuals who suspect exposure to HIV, delay testing up to one to four years.^3–5^ Annually, of those who test positive for HIV in the US, 25%−33% fail to return for initial test results—presenting a major barrier to HIV prevention, education, and subsequent treatment. Alarmingly, the average length of time from testing HIV positive to entry into care ranges from one to five years.^6–9^ African Americans account for nearly 50% of the newly estimated HIV/AIDS diagnoses, with the majority being tested only after developing symptoms of AIDS.^1,10^ Data suggests that African Americans may be testing at higher rates than other ethnic communities. For instance, results from the 2006–2010 National Survey of Family Growth (NSFG) of HIV testing (outside of blood donation), found that while the proportions of those ever tested for HIV were similar for Hispanic and non-Hispanic White persons (approximately 60% among women and 40% among men), a higher percentage of non-Hispanic Black women (75%) and non-Hispanic Black men (61%) had ever been tested for HIV. Unfortunately, African Americans are more likely to postpone medical care after finding out that they are HIV positive.^11^ Shortening this time lag between diagnosis and initial treatment is crucial because antiretroviral medications are most effective when initiated in HIV infected individuals with a CD4 cell count >350.^12^

In order to successfully address the National HIV/AIDS Strategy (NHAS)^13^ and Healthy People 2020 HIV objectives, greater proportions of people living with HIV need to achieve each step across the HIV Care Continuum.^14–16^ There is a need to diagnose people living with HIV and link them to care within three months.^14–16^ There is also a need to engage and retain them in care, prescribe them antiretroviral therapy and help them become virally suppressed (HIV viral load <200 copies/mL).^14–16^ However, this can only be achieved by getting people tested for HIV in order to diagnose those who are living with HIV but unaware of their status.

In 2004, the introduction of salivary rapid testing (SRT), a rapid HIV test using a saliva sample made immediate results possible in community-based settings, thus eliminating the need for invasive techniques, lengthy delays for test results, and the need to return to the agency.^17^ Rapid tests may reduce the cost of testing and potentially increase: 1) the availability of testing funded by public agencies, 2) the effectiveness of screening high risk populations who infrequently seek care, 3) the total number of individuals who get tested and receive post-test counseling, and 4) early entry into care.^18,19^ The impact of such immediate availability on one’s likelihood to be tested and enter care has not been described.^20,21^

The purpose of this study was to describe African Americans’ likelihood of using SRT methods and entry into healthcare if HIV positive.

### Theoretical Perspective

**Figure 1:**
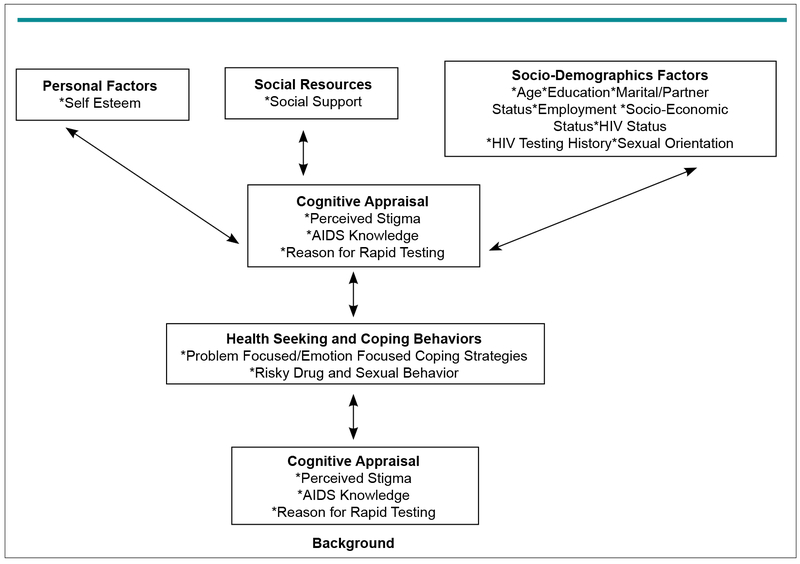
Modification of Comprehensive Health Seeking and Coping Paradigm (Modified with Permission).

The modified Comprehensive Health Seeking and Coping Paradigm (CHSCP)^22^ (Figure 1), framed this cross-sectional, exploratory study. Formative qualitative research using focus groups and cognitive interviewing were used to identify and describe the factors in CHSCP. The CHSCP—a model for health seek ing and coping developed by Nyamathi et al^22^—provided the specificity necessary to examine factors that may impact the likelihood of participating in SRT and adopting appropriate protective health behaviors such as obtaining healthcare if HIV positive. Nyamathi et al tested and refined the CHSCP in studies that examined HIV testing in the context of health seeking and coping in drug-abusing, homeless, and impoverished men and women.^23–29^ The current study was also guided by components of the Schlotfeldt Paradigm of Health Seeking Behaviors^30^ and the Lazarus Theoretical Schema of Coping and Adaptation.^31^ While the original CHSCP model used 12 factors, the modified version of the model (Figure 1) is composed of six factors (i.e., personal, coping resources, socio-demographic cognitive appraisal, health seeking and coping behaviors and health outcomes).

## BACKGROUND

### HIV Testing Types and Guidelines

The Centers for Disease Control and Prevention (CDC) recommends routine HIV screening in healthcare settings to allow earlier detection of HIV and to link patients with unknown HIV infection to clinical services.^33^ Routine HIV screening involves laboratory-based HIV testing procedures and point-of-care rapid HIV tests, such as SRT.^33^ One study that explored patient perspectives of rapid and routine HIV-testing in an urban urgent care center found that rapid HIV-testing was preferred to enzyme immunoassay (EIA) testing, primarily due to not having to return to the agency for results and reduced stress.^34^ Similar findings have been reported among African Americans and others who might otherwise refuse or avoid testing.^35–40^

However, the availability of non-invasive HIV testing does not always predict testing participation. Brown^42^ offered salivary testing to African Americans after participating in HIV prevention and reported a low participation rate (5.5%−23%). In 2005, the CDC assessed prevalence of HIV using rapid testing methods among men who have sex with men (MSM) in five large metropolitan areas and many (64%) of the men infected with HIV who were unaware of their status were African American.^43^

### Psychosocial Covariates of HIV Testing and Linkage to HIV Care

A synthesis of published studies regarding psychosocial factors associated with the decision to be tested for HIV showed that testing for HIV was more likely when individuals perceived that they have been at risk and/or perceive testing to be beneficial.^50^ They also identified fear of the consequences of testing positive —mainly worries related to discrimination and rejection—as major barrier to HIV testing. The CDC multi-state HIV Testing Surveys (HITS) conducted from 1996–2002^45,46^ and previous versions of the HITS^47,48^ revealed that the common reasons for not being tested included thinking exposure to HIV was unlikely, belief that they were HIV negative, and being afraid of testing positive. Other researchers have identified stigma (related to HIV or identifying as gay or MSM), self-esteem, social support as predictors of HIV testing.^34,51^

Daniels^35^ asserts that factors affecting testing in the African American community have more to do with testing circumstances such as being stigmatized for seeking an HIV test as opposed to access. Generally, racial and sexual discrimination towards HIV testing in African Americans affects health-care utilization, treatment adherence, and blocks access to essential treatment.^52–56^ Enhanced self-esteem has been (a) linked to greater testing and return for results,^23,24,73^ and (b) found to be a predictor of HIV risk behavior,^28^ and health seeking behaviors.

In one study, higher social support, HIV/AIDS knowledge, and perceived risk for HIV/AIDS scores were predictive of HIV testing and return for results.^23^ Social support is a strong predictor of healthcare use patterns^63^ and the decision to seek care if HIV infected. Lack of social support has also been found to result in inadequate access to healthcare, poorer continuity of care and non-adherence to medical appointments.^64–67^ Conversely, greater social support has been linked to increased acceptance of HIV testing and return rate for test results among high risk and minority populations.^72^ Knowlton, Hua, and Latkin^68^ found significant relationships between medical service use and social support networks, including greater sources of emotional, financial, and physical support in HIV infected drug users. Thus, when used in combination with an oral HIV test, social networks might increase testing rates in minorities.^68^

HIV testing is a necessary first step in establishing linkage to care, which is defined as having had at least one measurement of a CD4 level or one viral load test within 3 months after diagnosis.^85^ Hall et al.,^76^ using the Medical Monitoring Project (MMP) data from CDC and face-to-face interviews, reported that the following factors inhibited and decreased linkage to care: low rates of HIV testing, a reduced sense of urgency about the need for care, lack of health insurance, competing child care responsibilities, lack of transportation, lack of social support, poverty, food insecurity, unstable housing, unemployment, lower education level, homelessness, racism, stigma, homophobia, and mental health or substance abuse problems.

Little is known about the coping strategies used by people who enter the healthcare system shortly after an initial diagnosis of HIV infection. However, evidence shows that people with HIV who consistently seek care and adhere to clinic appointments use active (problem-focused) and avoidance (emotion-focused) coping mechanisms.^77^ Problem-focused coping strategies, as opposed to emotion-focused coping, have been associated with greater likelihood of HIV testing.^9,71,78,79^ Inhibited coping on the other hand, has been shown to predict greater non-adherence to clinic visits even after controlling for confounding effects of recent depressed mood.^80^

### Socio-Demographic Characteristics and HIV Testing and Treatment

Several studies report the effects of socio-demographic factors on HIV testing rates. Marital stability, low income, less education, ethnicity (African American), and less knowledge about testing have been shown to influence the likelihood of HIV testing.^41,82,83^ Researchers found that lack of interest in HIV testing was associated with being African American, female, older in age, being abstinent or without a partner, and having poorer knowledge of HIV.^82^

Variables related to seeking HIV care are ethnicity, having a CD4 cell count <200, identifying as MSM or heterosexual, injection drug use, and insurance status. In one study, being African American and/or Hispanic was associated with significantly lower knowledge about antiretroviral therapy, which has implications for entry into care if HIV positive.^74^

## METHODS

This cross-sectional, qualitative study was conducted using focus group methodology. The study aims were to (1) describe personal factors, social resources, socio-demographic factors, cognitive appraisal, and health and coping behaviors which predict or influence the likelihood of African Americans’ participation in salivary rapid testing (SRT) and, if positive, subsequent entry into healthcare. A structured interview guide and cognitive interviewing were used to conduct five focus groups (N=40) of African Americans recruited from an sexually transmitted infection (STI) clinic.

### Procedures

A large health department STI clinic in downtown Indianapolis served as the setting for the study. Individuals were eligible to participate in the focus groups if they designated their racial background as African American or Black. Other inclusion criteria included: age 18 years or older, able to read and write at a minimum of an 8^th^ grade reading level, and self-report of not having been tested for HIV prior to their clinic appointment. A screening survey was used to identify participants who met the specified criteria. Flyers and posters describing the purpose of the study, measures, inclusion/exclusion criteria, monetary incentives, enrollment process, and contact information were posted in visible areas of the STI clinic. Clinic staff were provided inclusion/exclusion criteria and given instructions on how to inform eligible participants of the study. Prospective participants contacted the researcher who scheduled a time for the focus group. Focus groups were conducted at the clinic immediately after they were discharged from the clinic and provided consent to participate. A $50 incentive was given to participants.

The investigator used a semi-structured interview guide (SSIG) to direct the focus groups. A trained research assistant operated the tape recorder and took notes. Questions and probes assessed knowledge about HIV infection, testing methods, their own perceived barriers and facilitators to HIV testing and entry into care if necessary, perceptions about HIV risk behaviors in their community, and possible ways to increase HIV testing with the SRT.

### Qualitative Data Analysis

Thematic content analysis was initiated by conducting a line-byline analysis of the focus group transcripts. The SSIG interview guide was used to structure to organize the data and three independent coders were used to assess inter-coder reliability. The principle investigator checked the transcripts against the audio-tape and made corrections as necessary. Sentences and phrases were reviewed for pattern recognition and/or core meanings. Recognized patterns were refined and synthesized into descriptive statements of the data provided by the participants. At the conclusion of the initial analysis, the team noted subtle differences in responses between men and women. Using the same procedures as in the initial analysis, an additional sub-analysis of the transcripts was conducted focusing on responses from women as compared to men.

Independent coders were used in the coding process. Transcripts were coded independently, and any disagreements were resolved through discussion. Finally, trustworthiness, credibility, transferability, dependability, and confirmability of the data were ensured by data verification ascertained by both research team members and participants.

An iterative process of comparison was used to further analyze the data, moving between individual elements of the text specific to participant responses to each of the three questions in the structured interview guide. Several cycles of comparison were accomplished: across all accounts to identify particular themes, subthemes, categories, and classes; within each individual account to identify meanings that were implicit rather than explicit in the text; and of one whole account with another to identify overall patterns of meaning.^86^

## RESULTS

### Participants

Of the 38 African American adults who participated in the study, 16 were female between the ages of 18–49 and the mean age was 23 years. Ten reported their marital status as single and six as partnered. There were 22 males, ages 18–49 and the mean age was 29.5 years. Fifteen reported their marital status as single, five as partnered and two as married. All self-identified as heterosexual with most reporting low income and no health insurance.

Eight themes emerged from the data: *familiarity (with HIV testing and HIV risk)*, *stigma (of HIV)*, *fear (of HIV)*, *access (to HIV testing)*, *immediacy of knowing (HIV test results)*, *ease of testing*, *degree of responsibility*, and *trust* (Table 1). Gender specific themes were *health maintenance* (women) and *illness management* (men). Sub-themes within gender-specific themes were *stoicism* (women) and *anger* (men). Each theme was not exclusively a barrier or facilitator, but was considered as one or the other depending on the aspect of HIV testing being discussed.

### Familiarity

Familiarity with HIV testing methods, particularly the new methods was perceived as being a facilitator to HIV testing. Lack of familiarity tended to be perceived as a barrier to testing. Participants felt that there were many people who had not been tested because they lacked knowledge about HIV and HIV risk. Most of the participants were knowledgeable about blood and saliva testing, but few were familiar with rapid testing methods. Some perceived this as something that would help more people to be tested, while others perceived it to be *a positive way to approach testing or they wondered if the rapid tests were less accurate since it took less time.* Others felt strongly that there should be more education about HIV risk and testing.

One participant said, *“Basically people are not getting tested because they’re not educated about it.*” The importance HIV knowledge in the management of the condition was also noted by a participant who observed that *“if you were positive, you’d really want information about how you can deal with the situation, or the pills you have to take, the appointments you need.*” However, there was evidence of lack of HIV knowledge especially that related to testing. A participant noted, “*I know you can give blood, but saliva, that’s something I never even heard of*”. Another acknowledged to insufficient knowledge on various ways one can be infected: “*I know you can get it through semen and things like that, but that’s the only ones I know of.*”

### Fear/Stigma

Most participants expressed the theme of “fear” or “stigma” of HIV. This is illustrated by the following participant comment: *“I think [that when] most people think about HIV or AIDS, the stereotype is gays. People think gays are people who started the epidemic or whatever, keep it spreading, although I’m sure that most gay people have it, but that would be a stigma to think about*.” Another participant said, “*everybody [would say] she’s over there doing that stuff, that HIV test. I would not even go near no HIV testing.*”

### Access to Testing

Most participants discussed the issue of accessibility and locale. One participant said, “*In the black community, like schools or churches, you could network with them probably, and people probably wouldn’t be so scared to walk into a church and get it done or go into a school to get it done, because everybody is not watching you, you know, you’re not so self-conscious about that.*” Similarly, another participant said, “*I think if they had a third spot to make it easier for people to go to, where they just go in, get swabbed and find out then a lot more people, in this downtown area, in the neighborhood, I think they would prob ably jump on it faster than trying to walk up in the clinic, and waiting, and getting it drawn, a piece of mind for their self.*”

### Immediacy of Knowing

Several participants had both negative and positive reactions to the rapidity of results. To those who had negative reactions, blood specimens and conventional HIV testing were viewed as “more accurate” than specimens obtained *via* a salivary rapid test. One person shared, *“Just a lot of times when things happen quickly there is a lot of chance of error, just from being so fast. Usually when things take more time then things are more detailed and organized.”* Another person expressed that, “*Even though it hurt* [the needle stick] *the blood, it seems like it would be more accurate than saliva”.* One participant described it in the following manner, “*“I would choose the rapid test because I would want to know right then instead of waiting around for them one or two weeks just moping, wondering whether or not.”* The relief associated with not having to wait for prolonged periods of time was expressed by several participants. For example, one participant shared, “*I thought I’d do the rapid test because you’re going to know. You don’t have to sit and wait and wonder and then you get your results back and you find out yea or nay…”*

### Ease of Testing

Ease was discussed in terms of method (needle stick *versus* oral swab) used to obtain the specimen. Preference was given to oral swab, as needles evoked feelings of fear. One participant described it in the following manner, *“I’m thinking it would be much easier, because most times, African-Americans, black women go to doctors more than black men, so they’re scared of needles, scared the doctor don’t touch them and all that”.*

### Degree of Responsibility

Degree of responsibility was expressed in terms of degree of responsibility to self and degree of responsibility to a partner or new sexual contact to get tested. Responsible behaviors were described as learning one’s status in an effort to either learn prevention strategies to stay negative or “be safe”, or if positive, to prevent spreading the disease to others. Irresponsible behaviors were described as purposeful acts or attempts to infect others. One participant described it in the following manner, “*You’ve got people that think like, if I’ve got it, then I’m going to give it to other people, just because. It’s all about the mindset and how you take it.”* Participants felt that the manner in which one copes with having HIV influenced their degree of responsibility to protect themselves and others from the threat of HIV. One participant explained it as such, “*Some people, if they knew they was going to die, they’d probably go on and do all types of things illegal, if they definitely going to die.”*

### Trust

The majority of participants discussed the importance of trust. Factors that influenced trust included privacy and knowledge of the person providing counseling and testing. Residential settings, such as apartment complexes were preferred over testing in facilities that were distinctively known as an HIV testing site. Non-traditional sites were favorable in they offered convenience and privacy. One participant described it as follows, *“I think it would be better or beneficial if you could go to them, maybe go outside an apartment complex or something, and just do it confidentially, but go to them, so you won’t have to find transportation and find babysitters or something like that.”* One respondent had this to say about seeking HIV testing at a traditional testing site, *“That would be a bad thing, because everybody she’s over there doing that stuff, that HIV test. I would not even go near no HIV testing. It would have to be in a hospital or something.”*

### Gender-Based Themes

#### Health maintenance/stoicism (women):

When asked to describe factors or situations that would facilitate HIV testing, one female respondent indicated the following, “*For my health really. I mean you should get it often just to be safe and make sure everything is okay. I mean, I don’t know why people wouldn’t get it done*”. This proactive approach is characteristic of problem-focused coping and is commonly used in situations where one feels optimistic about the outcome or feels as though something constructive can be done to eliminate the problem. The need to know one’s status to order to alleviate worry was commonly expressed by some women in the study. One woman said, “*If I had it and I needed help, that is one more reason for me to get it [the test]. I wouldn’t be scared of what people think, and whatever they think, it don’t make my decision no different*”. Some women did not perceive that men held the same beliefs and practices as women. One female participant said, “*Girls go to the doctor if they sneeze wrong. Men, if they sneeze wrong, they gonna keep on sneezing wrong until they start sneezing right*”.

#### Illness management/anger (men):

HIV testing emerged as a reactive behavior to a potential threat of illness, among male participants, more so than females. Unlike the majority of female participants, who viewed HIV testing as a proactive behavior necessary to ensure maintenance of health, most male participants viewed it as necessary if they had been careless. The following quote illustrates it best, “*If you just have a real high sex life, like a whole bunch of partying, and you get to thinking, and you don’t want to harm nobody*”.

Comparatively, men who reported being in a monogamous relationship expressed that they did not feel the need to get tested. One participant said, “*If I was having sex with a partner or different people I would want to get tested at least once or twice a year, but I’m not doing that anymore. I’m only with one person, and we know our status and everything, so we just keep it like that. But I still use condoms*”.

When asked about the emotional response to an HIV diagnosis, several men in this study responded that they thought that most men would respond negatively. For example, one male participant said, “*they [other men] would be pretty mad to find out that they got HIV, and try to take it out on everybody else in the surroundings*”. Similar comments were expressed by another male participant who said, “*They might be thinking I don’t want to go down by myself. So they might go try to back to everybody they took down*”. These responses are characteristic of emotion-focused coping responses, which may manifest as continued engagement in risk behaviors, denial and pessimism.

## DISCUSSION

This qualitative study identified eight themes regarding perceptions of HIV testing, specifically, SRT and subsequent entry into HIV care if positive in a sample of African Americans. Perceived risk and knowledge of HIV transmission have been noted to be predictors of seeking HIV testing.^23,24^ Similar to other studies, participants in the current study also noted the importance of HIV knowledge in testing. Given recent literature showing a connection between lack of correct HIV knowledge and low-level of HIV test uptake and risky sexual behaviors,^87^ their need for programmatic efforts to increase such HIV related knowledge especially in the African American community. Furthermore, it appears that some of the low-levels of follow-up testing can be attributed to lack of knowledge. While SRTs have the potential to increase the level of testing, educating the community about the test is important if it is to gain acceptance.

Similar to the findings in this study, HIV stigma and discrimination have been well-documented in the literature.^52,56,59,88–91^ Deacon,^88^ in a review of HIV stigma literature noted that the fundamental cause and mechanism of stigma stems from blaming certain groups of people for having an illness which provides an opportunity for those with stigmatizing attitudes to distance themselves and their in-groups from risk of infection. The consequences of HIV stigma including acting as a barrier to uptake of HIV testing and treatment services has been documented throughout the literature.^89–91^ Stigma may also be associated with fear and rejection.

Fear of being HIV positive and of the social consequences, such as rejection by loved ones, loss of employment or housing, fear discrimination and violence has negative effect on the likelihood of test seeking.^43^ This concurs with Rudy’s^59^ observation in a study involving African American and Latino women at risk of HIV that stigma attached to HIV and the perception that they were engaging in risk behaviors influenced the women’s decision to a clinic to receive an HIV vaccine. Taking the test in an area where one is unknown appears to be one avenue individuals use to deal with perceived stigma.

There is, thus, a need for continued efforts to find innovative ways to eliminate barriers to both felt and perceived stigma. Some participants suggested neutral places like schools or churches as places some people may be more willing to go to get tested. In addition to finding ‘neutral’ places that participants are willing to take the test, there is need to advocate for social support, especially among those who may be positive. Previous literature has shown that social support is associated with increased acceptance of testing and return rate for test results among high risk and minority populations.^29,65^ However, for this strategy to be effective, privacy and confidentiality is of paramount importance.

Some participants had both negative and positive reactions to the rapidity of results. To those who had negative reactions, blood specimens and conventional HIV testing were viewed as “more accurate” than specimens obtained *via* a SRT. While the rapidity of the test results was a deterrent to SRT, it was favorable to others. Some aspects of these results are similar to findings from Hutchins et al^34^ who found that rapid HIV-testing was preferred to enzyme immunoassay (EIA) testing. In the current study, the immediacy of the results and not having to wait for long periods of time were facilitators to SRT. Similar findings have been reported among African Americans and other groups who might otherwise refuse or avoid testing.^35–40^ Overall, the data suggests that the immediacy of results may not be feasible for all African Americans.

Coping did not emerge as a major theme in the current study. However, many participants expressed that the manner in which one copes with having HIV influenced their degree of responsibility to protect themselves and others from the threat of HIV. Many of the participants’ responses were characteristic of emotion-focused coping responses, which may manifest as continued engagement in risk behaviors, denial and pessimism. These finding are consistent with coping strategies that have been identified in the literature within the context of HIV testing, which include problem-focused coping and emotion-focused coping.^23^ According to the literature, African Americans tend to use more emotion-focused coping, an emotional response that occurs when people believe a situation to be hopeless or feel that it must be endured.^23^ Problem-focused coping, on the other hand, is used when people believe that something constructive can be done to alleviate a problem and has been used to predict acceptance of HIV testing and likelihood to return for test results.^9,71,78,79^ Overall, the literature illustrates the role of coping in acceptance of SRT.

Trust was an important theme for the majority of participants. This indicates that this population may be more inclined to seek HIV testing when it is offered in non-traditional settings. It may also suggest that in the absence of non-traditional testing sites, African Americans may not seek or consent to testing until they are hospitalized, at which point they may have advanced HIV disease and have fewer options for treatment.

These findings support the need to assess barriers and facilitators to HIV testing decisions in order to increase testing rates. The themes suggest the need for tailored community based interventions that decrease fear and stigma associated with HIV screening and increase trust in testing methods and providers.

### Implications for HIV Prevention and Research

Limited data is available describing factors impacting HIV test seeking, particularly SRT, or subsequent entry into care. Similarly, few if any cultural or population-based instruments are available to measure those factors in the context of this new non-invasive rapid testing modality. Therefore, this review addressed the concept of general HIV rapid testing and entry into care if needed. Based on the literature, there is evidence of initial support for relationships between the variables of the modified CHSCP and the study variables—likelihood to be tested with a saliva rapid HIV test and care entry if HIV positive. Some studies have examined the psychosocial, cognitive and behavioral variables in the context of HIV testing and entry into care. Other than the studies conducted by Nyamathi et al^22,23,25–28^ and Flowers et al^51^ none described these variables comprehensively. Studies similar to those conducted by Nyamathi and this current study support the development and testing of interventions to increase HIV testing rates and facilitate immediate or very early into the healthcare system as needed. The literature addressing variables associated with the likelihood of care entry indicates that African Americans are underrepresented in these studies.

In light of the newer salivary rapid HIV test method, there is a need to identify information addressing factors affecting the likelihood of HIV testing and subsequent entry into the healthcare system, if appropriate. The disparate number of African Americans infected with HIV must be examined in order to develop appropriate interventions. Also, to decrease disparity in care, research must be conducted that uses adapted or developed instruments appropriate and sensitive to the study population. Therefore, there is a crucial need to develop and test instruments to further examine African American’s HIV test seeking behaviors.

This review and study findings provide a foundation on which to base measurement of variables in a future study. Once tested, relationships shown among these variables can lead to the development of a psychosocial intervention to increase HIV testing using this newer salivary method. Additionally, social support and other interventions could be developed to increase testing of potentially HIV-infected people and subsequent entry into and retention in the healthcare system after the factors based on data from this and future studies. Identifying the factors that influence the likelihood of an individual to seek HIV testing will provide information on which to base development of interventions to facilitate testing eventually leading to earlier healthcare. This study may also have the potential to impact health policy development related to reporting and testing methods.

## Figures and Tables

**Table 1:** Themes and Exemplars of Supporting Data.

Theme	Exemplars of Supporting Data
**Familiarity with testing and HIV risk**	“I know it’s **blood, and I know it’s saliva**. I think that is the only ones. I know you can get it through **semen and things** like that, but that’s the only ones I know of, saliva and blood.” “I don’t know too much. I know you can give blood, but saliva, that’s something I never even heard of. I don’t know too much about it, but I want to learn.” “Like he said, a class or something. Basically people are not getting tested because they’re not educated about it.” “No, I’ve never heard of a follow-up test.” “A lot, like how it’s [testing] done and when will it be back, and just really everything.” “Especially if you were positive, you’d really want information about how you can deal with the situation, or the pills you have to take, the appointments you need.” “I thought it was just straight taking blood. I didn’t know about everything else.” “I think it should be mandatory too. You need to know as much about it, if you’re going to try this test, you need to know as much as possible about it, everything about it, everything.” “Since it’s quicker, people might think it might not be that accurate.” “Just a lot of times when things [tests] happen quickly there is a lot of chance of error, just from being so fast. Usually when things take more time then things are more detailed and organized.”
**Stigma of HIV** **Stigma encompasses ethnicity, sexual practices/orientation, health care provider and testing facility.**	“If you are African-American and another African-American tests you for it, you won’t be judged…” “See, if it’s positive I guess sometime, people are in denial. And they just going to continue, they just don’t want to admit that they have it, and they’ll continue to have partners.” “…I think most people think about HIV or AIDS, the stereotype is gays. People think gays are people who started the epidemic or whatever, keep it spreading, although I’m sure that most gay people have it, but that would be a stigma to think about. That would be something like, I hope nobody I’m messing with is like this.” “If I had a friend that had it, and he was my friend, I don’t think I would turn my back on him…Just because he has something, I don’t know, I just think you shouldn’t treat people like that.” I still say you. Just because there is a lot of stigma in the media about healthcare and qualified people…but like the way society is, they just make you believe you can go here and get better treatment or because it is a white physician, you’ll have better equipment and better things like that.” I think in the black community, like schools or churches, you could network with them probably, and people probably wouldn’t be so scared to walk into a church and get it done or go into a school to get it done, because everybody is not watching you, you know, you’re not so self-conscious about that.” “Not only yes they know, but okay, I don’t want anyone to know. I don’t want to tell.” “Me, personally, yes, because I don’t live here. I live almost an hour away from here. It’s not that I’m afraid I am positive, it’s just that I don’t want to be tested in my home town. Just being further away is more comfortable.” “It has to be done in private.” “That would be a bad thing, because everybody she’s over there doing that stuff, that HIV test. I would not even go near no HIV testing. It would have to be in a hospital or something.”
**Fear of having HIV** **Fear of having HIV was discussed in terms of not knowing how to emotionally cope with a diagnosis of HIV infection and the uncertainty of how to financially manage medical bills if HIV infected.**	“It could be good and bad. Because people probably can’t mentally prepare for the emotional, physical breakdown that they going to have if they do find that they have it…” “If you take the test and they tell you, you can walk out the door, but there’s somebody you can talk to at the time.” “…Because if you really did have it, then it’s so much, it’s like a gang of hospital bills. Just for the littlest thing. So that could have the opportunity to just make you not want to get tested.” Myself, I think the reason for people not taking the test as much is because of hard times…Most of the times you’re worried about how you’re going to pay your bills…Not to say it’s not important, but you think about other things.” “Taking out the formality makes it easier…But seeing the papers, it brings to your mind they’re really going to do this, and I’m signing this and they’re going to do this.” I think that’s a lot better, because a lot of times.they want to be tested but they don’t want to ask.” “The fear of not knowing and I’d want to make sure my health was up to par.”
**Access to testing**	“Well, that’s true too, but to say somebody don’t have, is unable to pay for it. What would they do then? You know what I’m saying, pay for it, the disease, what would they do then? They go ahead and continue the process or what? Denial, turned away, or what? You know what I’m saying?” “I think you’d probably get your best results by going to the schools. Asking kids if they want to take it.. Just go different places and ask people if they want to do it.” “I think if they had a third spot to make it easier for people to go to, where they just go in, get swabbed and find out then a lot more people, in this downtown area, in the neighborhood, I think they would probably jump on it faster than trying to walk up in the clinic, and waiting, and getting it drawn, a piece of mind for their self.”
**Immediacy of knowing**	“I would choose the rapid test because I would want to know right then. Instead of waiting around for them one or two weeks just moping, wondering whether or not.” “I would rather know that they are doing an HIV test on me, I don’t want you to just go ahead and do it. “Even though it hurt, but the blood, it seems like it would be more accurate than saliva.” Yes. I thought I’d do the rapid test because you’re going to know. You don’t have to sit and wait and wonder and then you get your results back and you find out yea or nay…” “Just to know you don’t got it. Especially if you’ve been messing around with a number of people.” “A good thing…because you don’t know unless you have it or something.”
**Ease (of getting the test)**	“I’m thinking it would be much easier, because most times, African-Americans, black women go to doctors more than black men, so they’re scared of needles, scared the doctor don’t touch them and all that. It would be much easier, just come in and swab your mouth, see if you’ve got HIV and then if you don’t.” (If I had an AA woman to do the test) “I would go to her, because I would feel more comfortable, because I would think that she would better relate to me, as far as being around, better than probably you could.”
**Degree of responsibility**	“It is a good thing to get prepared for, you can go to counseling before you can leave and find out that you might have it. At least you can know steps that you might need to take for prevention, to live a better and healthier life. At least you could know before you might find out you had it.” “I think it depends. There are some people that don’t care what harm they do everybody, and then the people that do care, they’re going to be chilling. It’s time to be by myself, just don’t mess with nobody, those with HIV, keep to yourself.” “I believe you’d probably want to get it just to know your status, make sure you’re safe, to know yourself and to protect other people.” “I think it would help people to be more safe and then you’ve got people that think like if I’ve got it, then I’m going to give it to other people, just because. It’s all about the mindset and how you take it.” “Some people, if they knew they are going to die, they’d probably go on and do all types of things illegal, if they definitely going to die.” “The point like if you are getting married or in a relationship with someone and you would just want to know. Or getting into a new relationship…You might both want to get tested.” “I think they’d be more cautious with it probably.” “Maybe like a new partner, and obviously you trust the person, but you don’t really know. Just maybe as a precaution.”
**Trust**	“I think it would be better or beneficial if you could go to them, maybe go outside an apartment complex or something, and just do it confidentially, but go to them, so you won’t have to find transportation and find babysitters or something like that.” “It wouldn’t matter the color of your skin. It would matter the knowledge you got of what you’re telling me about. I need to know that you know what you are talking about in order to persuade me.” “I’d rather have a signed consent form, where you give your permission before testing.” “I would take the counseling because it’s good to know what is happening, get explained about what is happening to you, if you don’t know. So in that case, if you had somebody there that did know more, and could give you some information I didn’t know.” “I think they need to still explain what the procedure is, and get a signature from somebody.”

## ABBREVIATIONS:

SRT: Salivary Rapid Testing
CHSCP: Comprehensive Health Seeking and Coping Paradigm
CDC: Centers for Disease Control and Prevention
EIA: Enzyme Immuno-assay
STI: Sexually Transmitted Infection
